# Underrepresentation of Adults and Older Adults With Disabilities in Behavioral Clinical Trials: A Scoping Review

**DOI:** 10.1093/geroni/igaa057.2215

**Published:** 2020-12-16

**Authors:** Susan Stark, Marian Keglovits, Sandra Espín Tello

**Affiliations:** 1 Washington University in St. Louis, St. Louis, Missouri, United States; 2 Washington University School of Medicine, St. Louis, Missouri, United States; 3 University of the Basque Country (EHU/UPV), San Sebastián, Galicia, Spain

## Abstract

A lack of evidence-based interventions for people aging with long-term physical disabilities exists. To examine the exclusion of people with disabilities in behavioral clinical trials, a scoping review was conducted. ClinicalTrials.gov was searched for interventional behavioral studies from the United States completed from 2008–2018, with results focused on adults (18–64) and older adults (65+). In total, 158 clinical trials were included. In 129 articles, health conditions were excluded 697 times. Seventy-one clinical trials excluded at least one health condition with strong justification, 11 with poor justification, and 115 without justification. There is strong evidence that people with disabilities are excluded from behavioral clinical trials, often without justification. To help close this gap, our presentation will discuss how translational research strategies, focused on adapting existing EB behavioral trials, can be used to increase the availability of interventions that address the needs of individuals aging with and into long-term disabilities. Part of a symposium sponsored by the Lifelong Disabilities Interest Group.

